# Risk factors for methicillin-resistant *Staphylococcus aureus* colonization in a level-IV neonatal intensive care unit: a retrospective study

**DOI:** 10.1017/ash.2023.482

**Published:** 2023-10-31

**Authors:** Julia Elzbieta Galuszka, Kim Thomsen, Jenny Dahl Knudsen, Rikke Louise Stenkjaer, Rikke Nielsen, Karen Leth Nielsen, Andreas Petersen, Barbara Juliane Holzknecht, Mette Damkjaer Bartels, Morten Breindahl, Lise Aunsholt

**Affiliations:** 1 Department of Neonatology, Copenhagen University Hospital, Rigshospitalet, Copenhagen, Denmark; 2 Department of Clinical Microbiology, Copenhagen University Hospital, Rigshospitalet, Copenhagen, Denmark; 3 Statens Serum Institut, Denmark; 4 Department of Clinical Microbiology, Copenhagen University Hospital, Herlev and Gentofte, Herlev, Denmark; 5 Department of Clinical Medicine, University of Copenhagen, Copenhagen, Denmark; 6 Department of Clinical Microbiology, Copenhagen University Hospital, Amager and Hvidovre, Hvidovre, Denmark; 7 Comparative Pediatrics and Nutrition, University of Copenhagen, Copenhagen, Denmark

## Abstract

**Objective:**

To identify risk factors associated with methicillin-resistant *Staphylococcus aureus* (MRSA) colonization in neonatal patients during an MRSA outbreak to minimize future outbreaks.

**Design:**

Retrospective case-control study.

**Setting:**

Level-IV neonatal intensive care unit (NICU) at Copenhagen University Hospital, Rigshospitalet, Denmark.

**Patients:**

Neonates with either MRSA or methicillin-susceptible *Staphylococcus aureus* (MSSA)

**Methods:**

Methicillin-resistant *Staphylococcus aureus*-positive neonates were matched with those colonized or infected with MSSA in a 1:1 ratio. The control group was selected from clinical samples, whereas MRSA-positive neonates were identified from clinical samples or from screening. A total of 140 characteristics were investigated to identify risk factors associated with MRSA acquisition. The characteristics were categorized into three categories: patient, unit, and microbiological characteristics.

**Results:**

Out of 1,102 neonates screened for MRSA, between December 2019 and January 2022, 33 were MRSA positive. They were all colonized with an MRSA outbreak clone (*spa* type t127) and were included in this study. Four patients (12%) had severe infection. Admission due to respiratory diseases, need for intubation, need for peripheral venous catheters, admission to shared rooms with shared toilets and bath facilities in the aisles, and need for readmission were all correlated with later MRSA colonization (*P* < 0.05).

**Conclusion:**

We identified clinically relevant diseases, procedures, and facilities that predispose patients to potentially life-threatening MRSA infections. A specific MRSA reservoir remains unidentified; however, these findings have contributed to crucial changes in our NICU to reduce the number of MRSA infections and future outbreaks.

## Introduction

Neonates admitted to a neonatal intensive care unit (NICU) are susceptible to colonization and infection by bacteria because of their low birth weight, long hospital stay, and the need for invasive procedures.^1–4^ Methicillin-susceptible *Staphylococcus. aureus* (MSSA) and methicillin-resistant *S. aureus* (MRSA) are among the most prevalent pathogenic bacteria found in NICUs.^1,3^ Although MRSA and MSSA both cause a variety of infections, ranging from mild skin infections to sepsis, the treatment of MRSA is more challenging because of its resistance to standard anti-staphylococcal β-lactam antibiotics.^5^ Although both MSSA and MRSA have been shown to cause outbreaks in NICUs, the focus has mainly been on MRSA outbreaks reported in various NICUs, with several risk factors investigated.^1,2,6–13^

Methicillin-resistant *S. aureus* may be transmitted directly from asymptomatic carriers (eg parents and health care professionals (HCPs)) or indirectly by contact with contaminated objects or environmental surfaces, where it can survive on dry surfaces for months.^14–16^ Close contact between admitted neonates and HCPs, parents, and the environment facilitates bacterial flora exchange and colonization, with a subsequent risk of infection.^1,2,4,17^

Denmark is considered to have a low MRSA prevalence with 1–2% methicillin resistance in *S. aureus*.^18,19^ Despite this, a unique outbreak of MRSA (*spa* type t127) has been ongoing since 2019 in our level-IV NICU at Copenhagen University Hospital, Rigshospitalet, Denmark. This study aimed to identify potential risk factors associated with MRSA colonization compared to neonates with MSSA with the purpose of enhancing the capability to reduce future outbreaks. This was accomplished by performing a multifactorial retrospective investigation, in which the patient, unit, and microbiological characteristics during admission to our NICU were identified and analyzed while concurrently evaluating and adapting the department’s handling of the outbreak.

## Materials and methods

This single-center retrospective study was approved by the Danish Patient Safety Authority (approval number R-21048862), without the need for parental consent. The study was conducted in a highly specialized level-IV NICU (defined by competencies in infant surgery, including cardiac surgery and extracorporeal membrane oxygenation) at Copenhagen University Hospital, Rigshospitalet, Denmark.

### Neonatal intensive care unit setting

Approximately 1,200 neonates between the gestational age of 23 weeks and 2 years of age are admitted to the NICU annually. The NICU employed 160 nurses, 30 physicians, and 20 staff. It is divided into two units: one for neonates with gestational age ≥ 34 weeks (mature) and the other for neonates with gestational age < 34 weeks (premature). The latter group of neonates had an average length of hospital stay of 73 days.^20^ There is a high level of collaboration between HCPs in the 2 units, and all shared areas are utilized by HCPs from both units. Health care professionals in our NICU include all employees in the department, including service and administrative staff, nurses, doctors, and leaders. All nurses can perform respiratory care for the neonates if needed. Seventeen rooms were dedicated to the 33 neonates, with one parental bed beside each neonate. Most rooms are shared. In 7 of the 17 rooms, parents had access to separate toilets and bath facilities; parents situated in the remaining rooms shared 2 toilets with bath facilities accessed from the aisles. Furthermore, all the parents shared 1 kitchen. Parents are actively involved in the daily caregiving of neonates with unrestricted access 24/7/365, using a family-centered care approach.

### Methicillin-resistant *S. aureus* screening in the capital region of Denmark

In the Capital Region of Denmark, neonates who were step-down transferred from the level-IV to the level-II NICU (defined as providing intensive care for sick and premature newborns who do not require mechanical respiratory care) were isolated at arrival and kept in isolation until negative screening results. Neonates transferred to the level-IV NICU were screened for MRSA upon arrival but were not isolated. This is explained by the high transfer activity, limited number of rooms, and shortage of staff. If needed isolation was initiated. In the case of a positive test result in a neonate after step-down transfer, all neonates at discharging level-IV NICU were screened for MRSA. If a MRSA-positive case was identified, screenings were performed weekly for 3 consecutive weeks. Longitudinal screenings were omitted in case of negative test results exclusively. Details regarding the screening are presented in Table 1.

**Table 1.** All initiatives initiated by the task force group consisting of health care professionals (HCPs) from the Neonatal Intensive Care Unit (NICU) and Department of Clinical Microbiology in the NICU during the outbreak period

Categories	Initiatives
Surveillance cultures from neonates (n = 1,102) admitted in the NICU from December 2019 to January 2022	Swabs were collected from the nose, pharynx, perineum, and other possible infection sites from all hospitalized neonates during hospitalization in the NICU or at admission from other units, hospitals, or homes. All swabs were analyzed at the Department of Clinical Microbiology.
Surveillance cultures x 4 from HCPs employed in the NICU between February 2019 to January 2022. March 2020 July 2020 March 2021 April 2021	Surveillance cultures were collected voluntarily from the majority of HCPs (including physicians from the NICU and other departments, nurses, medical students, biomedical laboratory technicians, medical secretaries, and cleaning staff). Swabs were collected from nose and pharynx performed by other HCPs. All swabs were analyzed at the Department of Clinical Microbiology. The outbreak strain, *spa* t127, was found in 2 HCPs in the surveillance cultures from March 2020. The percentage of HCPs tested: March 2020 = 99 %, July 2020 = 92 %, March 2021 = 43 %*, April 2021 = 95 %*
Line list (May 2020)	A line list was developed containing key information on each MRSA-positive patient.
Epidemic curve	An epidemic curve was constructed to understand the transmission and get an overview of the extent of the outbreak.
Flow diagram	A flow diagram was constructed to establish future surveillance and isolation procedures.
Environmental testing	Environmental tests were collected from: • Ventilation ducts • Electric breast milk pump machines • Incubators Place of the specific tests was decided based on dialog in the task force group. It was concluded that the mentioned places could be potential MRSA reservoirs.
Environmental cleaning and hygiene changes	The cleaning and disinfection procedures were evaluated in collaboration with the cleaning unit and the hygiene nurse. Following changes were implemented in the NICU environment: • Removal of cloth curtains and upholstered furniture • Implementing separate stethoscopes for each incubator/cradle • Implementing plastic aprons when in contact with patients • Encouraging closed doors to patient rooms • Installation of automatic doors with motion sensors in relevant rooms • Placement of pictograms with hand-hygiene information above all sinks • Implementing a cleaning checklist and surface disinfection with RHEA Compact machine after discharge of isolated families • Placement of separate electric breast milk pump machines next to each parent bed • The patient rooms were divided into family and HCP zones. • Access to general personal protective equipment (PPE) in patient rooms and critical areas was improved • Implementation of single-use utensils • Monitoring of hand hygiene
Equipment decontamination	An evaluation was performed to optimize the cleaning and decontamination procedures of incubators, cradles, X-ray aprons, electric breast milk pump machines, thermometers, stethoscopes, and more.
Communication with HCPs and families	Increased levels of MRSA-relevant communication were initiated to HCPs connected to the outbreak, hygiene nurses, and other neonatal departments to which the neonates could be transferred. Oral and written information was communicated to the families admitted to the NICU.
Education of HCPs	All HCPs were educated in correct handling and selection of isolation equipment. Education of correct hand hygiene was performed using a UV light box. Education in hygiene was encouraged by using quizzes, journal clubs with peer-reviewed MRSA articles, weekly plan-do-study-act meetings, and weekly newsletters.
Education and guidance of families	Parents and visitors were encouraged to increase levels of hand hygiene at the entrance to the unit and to the patient rooms. This was encouraged by placing stickers next to each disinfection dispenser and sink. An oral introduction to the hand hygiene procedures and cleaning of the patients was given to the parents by the HCP welcoming the parents. Bags with information about hygiene and cleaning were placed on each parent’s bed. Parents were not allowed in milk kitchens. Parents with a potential MRSA contamination were screened in advance including parents working on pig-farm, parents with a prior MRSA contamination, and parents with admission to a hospital in a foreign country.
Audits	The Department of Infection Control performed 2 audits concerning respiratory equipment and general hygiene in the department.
Collaboration with collaborating units	A monthly e-mail update about the MRSA outbreak was sent out to collaborating units.

* This screening was only performed on 1 of the nursing teams (mature) due to individual circumstances in of 1 of the patients in the team.

### Study design and data collection

We reviewed patient file information from neonates admitted to the NICU between December 2019 and January 2022. Of the 45 MRSA-positive neonates, 33 were MRSA-positive for *spa* type t127. All infants tested positive for MRSA were newborn NICU patients, and infants older than neonate, were not included in this study. Neonates of similar gestational age who were admitted in the same period with confirmed cultures of MSSA were used as matching controls in a 1:1 ratio. Methicillin-susceptible *Staphylococcus aureus* was selected as a control because of its similar microbiological characteristics and pathogenesis.

Demographic characteristics and variables were collected from the medical records of all neonates. The 140 investigated variables were divided into three subcategories: (1) patient, (2) unit, and (3) microbiological characteristics (including treatment with antibiotics and MRSA testing). The variables were selected based on variables and findings identified in previous studies.^6,12^

### Microbiology laboratory methods

At the Department of Clinical Microbiology at Rigshospitalet screening samples from each neonate were pooled for culture and inoculated in MRSA enrichment broth (Tryptic Soy Broth (TSB) containing 2.5% salt, 3.5 mg/L cefoxitin, and 20 mg/L aztreonam) for overnight incubation at 35^o^C. Methicillin-resistant *Staphylococcus aureus* DNA in the TSB culture was identified using the BD MAX StaphSR Kit, according to the manufacturer’s protocol (BD Diagnostics, Sparks, MD, USA). TSB cultures were plated on CHROMID® MRSA agar (bioMérieux Diagnostics, Marcy l’Etoile, France), and MRSA colonies were identified and confirmed by MALDI-TOF MS (Bruker Daltonik GmbH, Bremen, Germany). The presumptive growth of MRSA in the clinical samples was identified using MALDI-TOF MS. Subsequent MRSA DNA was detected using the Xpert® MRSA NxG test on the GeneXpert® Dx System (Cepheid, Sunnyvale, CA, USA). Nose, throat, and perineal samples from neonates transferred from the NICU at Rigshospitalet were tested at the Department of Clinical Microbiology at the Amager Hvidovre Hospital and Herlev Gentofte Hospital. Each sample was inoculated into MRSA enrichment broth (TSB containing 2.5% salt, 3.5 mg/L cefoxitin, and 20 mg/L aztreonam) for overnight incubation at 35^o^C and then plated on MRSA CHROMagar^TM^ (CHROMagar, Paris, France). Due to the elevated risk of false positive outcomes associated with the GeneExpert test, the department was advised to exclusively depend on targeted polymerase chain reaction testing throughout the course of this outbreak.

All isolates underwent antimicrobial susceptibility testing in accordance with the guidelines of the European Committee on Antimicrobial Susceptibility Testing.^21^ Thirty of the 33 samples were available for WGS performed at Rigshospitalet (n = 17), the National Reference Laboratory for Antimicrobial Resistance at the Statens Serum Institute (n = 5), or the Amager-Hvidovre Hospital (n = 8) on the MiSeq or NextSeq Illumina platforms. All raw reads were analyzed using Rigshospitalet with BacDist to determine genetic relationships, and nucleotide polymorphisms were determined using GenBank accession number NC_007795 as the reference genome.^22^ Multi-Locus Sequence Typing (MLST) types were determined with PubMLST from assemblies generated with Shovill using SPAdes.^23,24^

### Statistical analysis

Categorical and numerical data were summarized using the table-one package in R.^25^ The distribution of numerical variables was determined using the Shapiro test. All statistical analyses were performed using MSSA, as the control group. The least Angle Shrinkage and Selection Operator with k-fold cross-validation (*k* = 10) were used to select predictive variables. This was done due to the relatively small population and to eliminate the possibility of confounding variables. Predictive coefficients were determined based on a high number of coefficients relative to the sample size. Variables with only 1-factor level or fewer than 6 observations for at least 1 group were excluded from statistical analysis. Sub-variables (marked with italics in the Supplementary Material (S1)) were only included in further analysis if the parent variable was selected as a predictive coefficient by the Least Angle Shrinkage and Selection Operator. By performing Least Angle Shrinkage and Selection Operator it was possible to perform univariate logistic regression analysis with a 95% significance level for the 10 most predictive coefficients determined by the Least Angle Shrinkage and Selection Operator. Odds ratios were determined using univariate logistic regression analysis. Statistical analyses were performed using R software for Windows (version 4.1.2).^26^

## Results

Out of the 2,472 neonates admitted to the NICU during the study period, 1,102 were screened for MRSA. Of these, 33 cases (3%) were identified with a positive culture of MRSA (*spa* type t127) of which 4 developed a severe MRSA infection. Baseline MRSA infections were lower than national incidence of 1,6% in *S. aureus* bacteremia cases.

### Outbreak description

**Figure 1.**
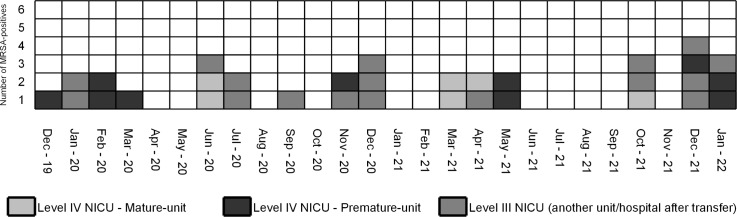
An epicurve of all 33 cases of MRSA in the NICU over the course of the study in months. Each cell represents 1 MRSA-positive neonate, and the cells are grayscale-coded after the place of identification of the MRSA.

The index case of the outbreak was identified as an isolate from the respiratory tract in December 2019. The outbreak was confirmed in January 2020 when 2 additional neonates had the same MRSA strains. Of the remaining 30 neonates, 17 were culture-positive upon transfer to another NICU. Cases were often adjacent to each other as shown in the epicurve of this outbreak in Figure 1.

The index neonate was hospitalized for approximately 2 months due to a congenital disease and the need for surgery. During admission, the neonate was critically ill and received numerous invasive interventions. The neonate died of the disease during admission.

### Infection cases

Four of the 33 neonates developed clinical infection. Three were hospitalized because of immaturity and developed sepsis with MRSA-positive blood cultures during admission. The 4th neonate was hospitalized due to respiratory and urogenital disease and developed pyelonephritis caused by MRSA. After discharge, the neonate was readmitted due to symptoms of sepsis. All 4 patients received intensive care, including respiratory support and antibiotic treatment.

### Outcome measures

Table 2 presents the main variables investigated in this study. An extended version of Table 2, containing sub-variables, is available in the Supplementary Material (S1). The predictive coefficients identified by statistical analysis are listed in Table 3.

**Table 2.** Main variables were collected from medical records of neonates with MRSA or MSSA. All variables were collected from time of admission to discharge from our Level-IV NICU. An extended version with sub-variables is available in Supplementary Material (S1). Categorical variables are summarized as absolute counts and percentages and numerical variables are summarized by their means and standard deviations or by their medians and interquartile ranges (IQR). Variables are divided into 3 categories (marked in dark gray) with subcategories (marked in light gray)

	MRSA	MSSA
n	33	33
Patient characteristics		
Gender = male (%)	18 (54.5)	20 (60.6)
Gestational age (weeks) (median [IQR])	31.29 [27.43, 34.29]	32.14 [27.29, 34.00]
Birth weight (g) (median [IQR])	1530.00 [943.00, 2420.00]	1650.00 [1020.00, 2200.00]
Underlying medical condition (%)		
* Cardiac = yes (%)*	9 (27.3)	5 (15.2)
* Respiratory = yes (%)*	14 (42.4)	2 (6.1)
* Gastrointestinal = yes (%)*	4 (12.1)	3 (9.1)
* Urogenital = yes (%)*	2 (6.1)	1 (3.0)
* Other = yes (%)*	4 (12.1)	1 (3.0)
Place of birth = inborn (Rigshospitalet) (%)	26 (78.8)	31 (93.9)
Length of stay (days) (median [IQR])	17.00 [7.00, 68.00]	28.00 [15.00, 40.00]
Number of admissions (median [IQR])	1.00 [1.00, 2.00]	1.00 [1.00, 1.00]
Cradle = yes (%)	28 (84.8)	27 (81.8)
Incubator = yes (%)	20 (60.6)	17 (51.5)
Mors (death) = yes (%)	1 (3.0)	2 (6.1)
Birth characteristics		
Birth method = vaginal (%)	13 (39.4)	11 (33.3)
Cesarean-section (%)		
* Category 1*	3 (15.8)	6 (31.6)
* Category 2*	8 (42.1)	4 (21.1)
* Category 3*	6 (31.6)	8 (42.1)
* Scheduled*	2 (10.5)	1 (5.3)
Twins or triplets = yes (%)	10 (30.3)	8 (24.2)
Feeding		
Intravenous alimentation = yes (%)	13 (39.4)	13 (39.4)
Mothers milk = yes (%)	32 (97.0)	32 (97.0)
Formula = yes (%)	12 (36.4)	8 (24.2)
Donor milk = yes (%)	24 (72.7)	22 (66.7)
Stomach tube = yes (%)	32 (97.0)	31 (93.9)
Breastfeeding = yes (%)	23 (69.7)	23 (69.7)
Skin perforations		
Drainage tube = yes (%)	6 (18.2)	1 (3.0)
Drainage placement (%)		
* Ascites*	0 (0.0)	0 (0.0)
* Cardiac*	2 (33.3)	0 (0.0)
* Pleura*	4 (66.7)	1 (100.0)
Number of drains (median [IQR])	1.00 [0.00, 2.75]	2.00 [2.00, 2.00]
PVC^a^ = yes (%)	32 (97.0)	22 (66.7)
Number of PVCs (median [IQR])	2.00 [1.00, 8.00]	2.00 [0.00, 3.00]
CVC^b^ = yes (%)	10 (30.3)	3 (9.1)
Number of CVCs (median [IQR])	0.00 [0.00, 1.00]	0.00 [0.00, 0.00]
UVC^c^ = yes (%)	15 (45.5)	10 (30.3)
Number of UVCs (median [IQR])	0.00 [0.00, 1.00]	0.00 [0.00, 0.00]
UAC^d^ = yes (%)	7 (21.2)	8 (24.2)
Number of UACs (median [IQR])	0.00 [0.00, 0.00]	0.00 [0.00, 0.00]
LL^e^ = yes (%)	11 (33.3)	10 (30.3)
Number of LLs (median [IQR])	0.00 [0.00, 1.00]	0.00 [0.00, 1.00]
Arterial cannulation = yes (%)	11 (33.3)	4 (12.1)
Number of arteria cannulations (median [IQR])	0.00 [0.00, 1.00]	0.00 [0.00, 0.00]
Capillary blood samples = yes (%)	33 (100.0)	33 (100.0)
Number of capillary blood samples (median [IQR])	8.00 [6.00, 23.00]	10.00 [7.00, 16.00]
Total number of skin perforations (median [IQR])	16.00 [7.00, 35.00]	13.00 [7.00, 22.00]
Surgery = yes (%)	12 (36.4)	4 (12.1)
Respiratory support		
Intubation = yes (%)	25 (75.8)	11 (33.3)
Respirator = yes (%)	19 (57.6)	9 (27.3)
* Respirator days (median [IQR])*	1.00 [0.00, 4.00]	0.00 [0.00, 1.00]
CPAP^f^ = yes (%)	28 (84.8)	25 (75.8)
* CPAP days (median [IQR])*	7.00 [2.00, 20.00]	4.00 [1.00, 11.00]
HF^g^ = yes (%)	14 (42.4)	12 (36.4)
* HF days (median [IQR])*	0.00 [0.00, 14.00]	0.00 [0.00, 4.00]
NIV^h^ = yes (%)	1 (3.0)	1 (3.0)
* NIV days (median [IQR])*	0.00 [0.00, 0.00]	0.00 [0.00, 0.00]
Total number of days in respiratory support (median [IQR])	10.00 [3.00, 32.00]	5.00 [1.00, 26.00]
Imaging		
ECG = yes (%)	6 (18.2)	3 (9.1)
X-ray = yes (%)	27 (81.8)	16 (48.5)
CT = yes (%)	2 (6.1)	0 (0.0)
Ultrasound (US) = yes (%)	15 (45.5)	22 (66.7)
MR = yes (%)	2 (6.1)	0 (0.0)
Echocardiography = yes (%)	15 (45.5)	8 (24.2)
ECMO^i^ = yes (%)	1 (3.0)	0 (0.0)
Department characteristics		
Number of fellow patients in room (median [IQR])	2.00 [1.00, 3.00]	2.00 [1.00, 5.00]
Number of HCP contacts (median [IQR])	42.00 [24.00, 86.00]	39.00 [28.00, 63.00]
Admitted to premature-unit first = yes (%)	11 (33.3)	2 (6.1)
Admitted to mature-unit = yes (%)	24 (72.7)	10 (30.3)
Admitted to premature-unit = yes (%)	19 (57.6)	24 (72.7)
Admitted to unit 7044 = yes (%)	4 (12.1)	2 (6.1)
Total number of rooms (median [IQR])	2.00 [1.00, 3.00]	1.00 [1.00, 2.00]
Microbiological characteristics		
Antibiotics		
Antibiotics during admission = yes (%)	23 (69.7)	15 (45.5)
Number of antibiotics administrated (median [IQR])	9.00 [0.00, 30.00]	0.00 [0.00, 15.00]
Number of different antibiotics (median [IQR])	2.00 [0.00, 4.00]	0.00 [0.00, 3.00]
MRSA-testing		
Hospitalization days before positive test (median [IQR])	3.00 [0.00, 20.00]	10.00 [5.00, 20.00]
Postmenstrual age at first test (weeks) (median [IQR])	33.10 [31.10, 38.30]	34.60 [31.20, 36.60]
Sampling site^j^ (%)		
Screening samples		
* Nose-throat-perineum* ^k^ *= yes (%)*	12 (36.4)	1 (3.0)
* Nose = yes (%)*	16 (48.5)	0 (0.0)
* Throat = yes (%)*	18 (54.5)	0 (0.0)
* Perineum = yes (%)*	15 (45.5)	0 (0.0)
Clinical samples		
* Wound = yes (%)*	3 (9.1)	3 (9.1)
* Sputum = yes (%)*	5 (15.2)	6 (18.2)
* Conjuctiva = yes (%)*	6 (18.2)	19 (57.6)
* Blood = yes (%)*	4 (12.1)	9 (27.3)
* Other = yes (%)*	9 (27.3)	4 (12.5)

a PVC = peripheral venous catheter.

b CVC = Central venous catheter.

c UVC = Umbilical venous catheter.

d UAC = Umbilical artery catheter.

e LL = Long line.

f CPAP = Continuous positive airway pressure.

g HF = High flow.

h NIV = Noninvasive ventilation.

i ECMO = Extracorporeal membrane oxygenation.

j Each neonate can be included several times if the neonate has been colonized in several places.

k These are pooled samples; that is, we do not know if the neonate is positive in 1, 2, or 3 places.

**Table 3.** The predictive coefficients selected by LASSO and *P*-values for these coefficients were determined by ULR for MSSA as control group for MRSA.

Characteristics	N	OR^a^	95% CI^b^	*P*-value
Patient characteristics				
Birthweight	66	1.00	1.00, 1.00	0.4
Underlying medical condition				
Respiratory	66	11.4	2.79, 78.0	0.003**
Cardiac	–	–	–	–
C-section category 2	–	–	–	–
Donor milk	–	–	–	–
Skin perforations				
PVC^c^	66	16.0	2.81, 303	0.010*
Number of PVCs	–	–	–	–
Respiratory support				
Intubation	66	6.25	2.21, 19.3	<0.001*
HF^d^ days	–	–	–	–
Department characteristics				
Admitted to mature-unit	66	6.13	2.18, 18.7	<0.001*
Admitted to premature-unit first	66	7.75	1.85, 53.4	0.012*
Number of admissions	66	4.25	1.40, 19.5	0.029*
Microbiological characteristics				
Number of antibiotics administrated	–	–	–	–

* Statistically significant results (*P* < 0.05) are marked with asterisks.

a OR = Odds Ratio.

b CI = Confidence interval.

c PVC = Peripheral venous catheter.

d HF = High flow.

### Patient and unit results

Having a respiratory underlying medical condition (UMC) was predictive of later colonization with MRSA when compared to MSSA (OR 11.4, 95% CI 2.98-78.0, *P* < 0.01). Respiratory UMC includes respiratory insufficiency, pneumonia, bronchitis, bronchopulmonary dysplasia, hydrothorax, and cystic fibrosis.

Furthermore, intubation during admission was predictive of later MRSA colonization (OR 6.25, 95% CI 2.21-19.3, *P* < 0.01), whereas other types of respiratory support were not selected as predictive coefficients for later MRSA colonization. Likewise, the application of peripheral venous catheters increased the risk for later MRSA colonization when compared with MSSA (OR 16, 95% CI 2.81-303, *P* < 0.05).

Admission to the premature unit (OR 7.75, 95% CI 1.85-53.4, *P* < 0.05) as the first unit was a predictive characteristic of later MRSA colonization when compared to MSSA. Admission to the mature unit at any time (OR, 95% CI 6.13, 2.19-18.7, *P* < 0.01) was also a predictive characteristic when compared with MSSA. Additionally, the number of admissions was generally predictive of later MRSA colonization when compared with MSSA (OR 4.25, 95% CI 1.40-19.5, *P* < 0.05).

### Initiatives in the neonatal intensive care unit

An interdisciplinary task force was established during this outbreak. It consisted of HCPs from the NICU and the Department of Clinical Microbiology. The group reviewed all cases of MRSA in the NICU with the hope of terminating the outbreak. All the outbreak control initiatives are described in detail in Table 1. The equipment (including the respiratory equipment) and patient rooms were inspected, and screening samples from the HCPs and environment were collected. This included ventilation ducts, electric breast milk pump machines, and incubators. No MRSA isolates were found in any environmental swabs, but two HCPs were positive for MRSA (*spa* type t127) in the second round of HCP screening.

### Microbiological results

**Figure 2.**
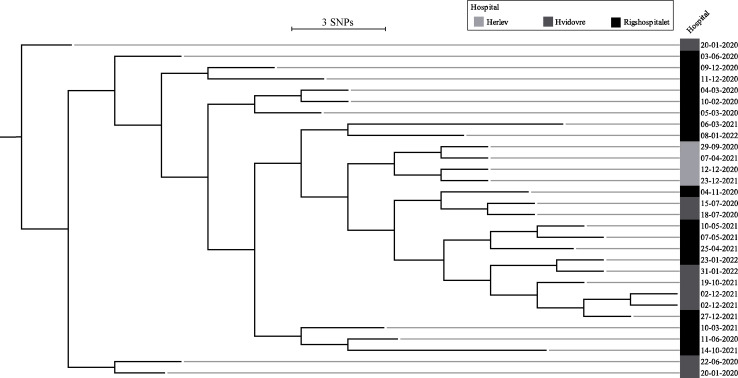
RAxML phylogenetic tree illustrating the genetic distance between 30 of the outbreak isolates presented in the context of which hospital they were collected from. The isolates are presented in chronological order (from top to bottom) from the beginning to the end of the study. The scale bar illustrates substitutions/sites.

Out of 33 MRSA-positive neonates, 17 were found by screening and were thus considered colonized and not infected. IOut of the remaining 16 neonates, MRSA was detected through clinically relevant testing due to suspected infection. Methicillin-susceptible *Staphylococcus aureus* was identified through clinically relevant testing exclusively. Phylogenetic analysis of the 30 MRSA isolates available for WGS revealed close clustering of all the isolates, thereby confirming the outbreak. The isolates were *spa* type t127, multi-locus sequence type 1 (ST1), and differed by only 1-24 SNPs, despite being collected over a two-year period (shown in Figure 2).

## Discussion/Conclusion

In this single-center retrospective study of an outbreak of MRSA t127, 33 neonates were colonized with MRSA, and 4 of these had an MRSA infection. Previous studies have documented the risk factors related to MRSA colonization in the NICU.^6,12^ However, these studies have mainly focused on patient-, unit-, or treatment-related factors separately, or investigated a few factors, limiting the overall understanding of MRSA as a multifaceted problem. Therefore, this retrospective case-control study collectively investigated all conceivable aspects by examining 140 variables related to patient, unit, and microbiological characteristics.

We found that neonates with respiratory UMC had a higher risk of later MRSA colonization. Respiratory UMC entails increased activity around the airway and the potential need for intubation. This increases the risk of exchange of bacterial flora with co-patients or HCPs and the risk of contaminating respiratory devices (e.g., Beneveniste Valve, laryngoscope, etc.). As MRSA can survive on dry surfaces for a longer period,^14,27^ suboptimal disinfection may result in an MRSA reservoir on the equipment or in the surroundings. It has been shown that neonates colonized with MRSA were more likely to develop an MRSA infection when exposed to mechanical ventilation for a longer period.^28^ However, in our study, there was no correlation with the total number of days of respiratory support.

Additionally, we found a correlation between later MRSA colonization and the insertion of peripheral venous catheters when compared to MSSA. Meanwhile, there was no correlation between the need for central venous catheters and subsequent MRSA colonization. One may speculate that establishing and maintaining peripheral venous catheters often necessitates multiple and repeated attempts during which the skin is perforated, especially in preterm neonates. There is no established procedure in the NICU for registering the number of attempts. In addition, central venous catheters are always placed during sterile procedures and are closely monitored using HCP.

Unsurprisingly, we found that frequent admission to rooms without separate parental toilets and bath facilities was associated with a greater risk of subsequent MRSA colonization. Shared facilities can potentially increase the possibility of an MRSA reservoir in the environment as parents could be MRSA-positive before isolation.

Numerous interventions were continuously implemented by an interdisciplinary task force group during the MRSA outbreak (Table 1). This involved practical changes in the environment, modifications to cleaning procedures, and revisions to specific protocols. Throughout the process, ongoing hygiene training was conducted, and the task force group delivered presentations and instructional sessions. An additional round of HCP testing was conducted where 2 HCPs tested positive, and they were both successfully treated for MRSA colonization. Health care professional screening also included staff from other departments that sometimes visited the neonatal ward, such as ophthalmologists but who had no positive findings for *spa* type t127. Furthermore, none of the environmental samples tested positive. A more comprehensive investigation of the environment was also conducted. The surgical facilities and the staff were not tested during the outbreak since the spa type was only found in neonates with a history of NICU admission. However, the purpose was to improve hygiene standards, potentially removing any reservoir, rather than to trace it. All interventions were implemented continuously to rapidly limit the outbreak why the impact of each intervention was undetectable.

During this outbreak, a specific MRSA strain was expected to be acquired mainly through HCPs and the environment, whereas MSSA strains could be acquired through family members. This may explain why the characteristics associated with the procedures and contact with HCPs were found to be risk factors for MRSA infection, although no concrete reservoirs have been identified.

This study had some limitations. In-house MRSA screening in neonates was performed irregularly. In case of a positive test in a neonate (often when transferred to a step-down unit), we initiated weekly screenings in the weeks following the identification of a new case. When all weekly screenings were negative for 3 consecutive weeks, the screenings were omitted. Therefore, some MRSA cases may have been missed and the outbreak prolonged. Additionally, the controls were neonates with clinical symptoms and subsequent positive cultures for MSSA, whereas 17 of the 33 MRSA-positive neonates were exclusively colonized. However, in a level-IV NICU, no neonates are considered healthy, and clinical samples are examined frequently, thus increasing the chance of finding most clinical cases with the 2 bacteria. Furthermore, the surfaces within the shared toilet facilities used by parents were not evaluated for MRSA, despite the relevance of this area for testing. We should have considered a quality improvement study early during the outbreak. This could potentially have reduced transmission of MRSA and disease in some neonates.

In summary, neonates with a respiratory UMC and the need for invasive procedures and respiratory support were at significantly increased risk of MRSA colonization during the outbreak. Furthermore, the risk increases with readmission, shared rooms, and toilet facilities, thereby indicating an environmental source. Despite the identification of several risk factors for MRSA colonization, new MRSA t127 cases were found after the study period, with the last known case in August 2022. As of December 2022, 44 neonates with the outbreak clone were identified without a new case 11 weeks prior to the study. This indicates that the source of MRSA in this level-IV NICU was not identified. The findings from this study have contributed to a series of crucial and fundamental changes in our approach within the department, with an increased emphasis on hygiene, particularly during invasive procedures, and maintaining a clean working environment. Furthermore, the results have facilitated a more in-depth analysis of HPC hygiene practices, leading to tailored educational initiatives based on these findings. Additionally, these findings have contributed to a more systematic testing approach with repetitive screening during outbreaks and efficient interdisciplinary cooperation. All measures may have limited the transmission of MRSA and other pathogens with the aim of avoiding harmful outbreaks of multidrug-resistant bacteria.

## Supporting information

Galuszka et al. supplementary material Galuszka et al. supplementary material

## Supplementary material

The supplementary material for this article can be found at https://doi.org/10.1017/ash.2023.482
