# Supplementary material for: Risk factors for methicillin-resistant Staphylococcus aureus colonization in a level-IV neonatal intensive care unit: a retrospective study
Source: Antimicrob Steward Healthc Epidemiol. 2023 Oct 31;3(1):e194. doi: 10.1017/ash.2023.482 (PMC10654989; doi:10.1017/ash.2023.482)
Supplement: Galuszka et al. supplementary material [file S2732494X23004825sup001.docx]

**Supplementary material (S1).** All variables collected from medical records from infants with MRSA or MSSA. Categorical variables are summarized as absolute counts and percentages and numerical variables are summarized by their means and standard deviations or by their medians and interquartile ranges (IQR). Variables are divided into three categories (marked in dark grey) with subcategories (marked in light grey).

|  | **MRSA** | **MSSA** |
| --- | --- | --- |
| n | 33 | 33 |
| Patient characteristics |  |  |
| Gender = male (%) | 18 (54.5) | 20 (60.6) |
| Gestational age (weeks) (median [IQR]) | 31.29 [27.43, 34.29] | 32.14 [27.29, 34.00] |
| Birthweight (g) (median [IQR]) | 1530.00 [943.00, 2420.00] | 1650.00 [1020.00, 2200.00] |
| Underlying medical condition (%) |  |  |
| *Cardiac = yes (%)* | 9 (27.3) | 5 (15.2) |
| *Respiratory = yes (%)* | 14 (42.4) | 2 (6.1) |
| *Gastrointestinal = yes (%)* | 4 (12.1) | 3 (9.1) |
| *Urogenital = yes (%)* | 2 (6.1) | 1 (3.0) |
| *Other = yes (%)* | 4 (12.1) | 1 (3.0) |
| Place of birth = inborn (Rigshospitalet) (%) | 26 (78.8) | 31 (93.9) |
| Length of stay (days) (median [IQR]) | 17.00 [7.00, 68.00] | 28.00 [15.00, 40.00] |
| Number of admissions (median [IQR]) | 1.00 [1.00, 2.00] | 1.00 [1.00, 1.00] |
| Cradle = yes (%) | 28 (84.8) | 27 (81.8) |
| Incubator = yes (%) | 20 (60.6) | 17 (51.5) |
| Mors (death) = yes (%) | 1 (3.0) | 2 (6.1) |
| Birth characteristics |  |  |
| Birth method = vaginal (%) | 13 (39.4) | 11 (33.3) |
| Cesarean-section (%) |  |  |
| *Category 1* | 3 (15.8) | 6 (31.6) |
| *Category 2* | 8 (42.1) | 4 (21.1) |
| *Category 3* | 6 (31.6) | 8 (42.1) |
| *Scheduled* | 2 (10.5) | 1 (5.3) |
| Twins or triplets = yes (%) | 10 (30.3) | 8 (24.2) |
| Feeding |  |  |
| Intravenous alimentation = yes (%) | 13 (39.4) | 13 (39.4) |
| Mothers milk = yes (%) | 32 (97.0) | 32 (97.0) |
| Formula = yes (%) | 12 (36.4) | 8 (24.2) |
| Donor milk = yes (%) | 24 (72.7) | 22 (66.7) |
| Stomach tube = yes (%) | 32 (97.0) | 31 (93.9) |
| Breastfeeding = yes (%) | 23 (69.7) | 23 (69.7) |
| Skin perforations |  |  |
| Drainage tube = yes (%) | 6 (18.2) | 1 (3.0) |
| Drainage placement (%) |  |  |
| *Ascites* | 0 (0.0) | 0 (0.0) |
| *Cardiac* | 2 (33.3) | 0 (0.0) |
| *Pleura* | 4 (66.7) | 1 (100.0) |
| Number of drains (median [IQR]) | 1.00 [0.00, 2.75] | 2.00 [2.00, 2.00] |
| PVC^1^ = yes (%) | 32 (97.0) | 22 (66.7) |
| *Left upper extremity (median [IQR])* | 1.00 [1.00, 3.00] | 2.00 [1.00, 2.00] |
| *Left lower extremity (median [IQR])* | 2.00 [1.00, 2.00] | 2.00 [1.00, 2.00] |
| *Right upper extremity (median [IQR])* | 1.00 [1.00, 3.00] | 2.00 [1.00, 3.00] |
| *Right lower extremity (median [IQR])* | 1.00 [1.00, 2.00] | 1.00 [1.00, 1.00] |
| *Other (median [IQR])* | 1.00 [1.00, 1.00] | 1.00 [1.00, 1.00] |
| *Unknown (median [IQR])* | 1.00 [1.00, 1.00] | 1.00 [1.00, 1.00] |
| Number of PVCs (median [IQR]) | 2.00 [1.00, 8.00] | 2.00 [0.00, 3.00] |
| CVC^2^ = yes (%) | 10 (30.3) | 3 (9.1) |
| *Vena jungularis interna (median [IQR])* | 1.00 [1.00, 1.00] | NA [NA, NA] |
| *Vena jungularis externa (median [IQR])* | 1.50 [1.25, 1.75] | 1.00 [1.00, 1.00] |
| *Vena femoralis (median [IQR])* | 1.00 [1.00, 1.00] | 1.00 [1.00, 1.00] |
| *Vena subclavia (median [IQR])* | 1.00 [1.00, 1.00] | NA [NA, NA] |
| *Vena brachiocephalica (median [IQR])* | NA [NA, NA] | NA [NA, NA] |
| *Unknown (median [IQR])* | 2.00 [2.00, 2.00] | 1.00 [1.00, 1.00] |
| Number of CVCs (median [IQR]) | 0.00 [0.00, 1.00] | 0.00 [0.00, 0.00] |
| UVC^3^ = yes (%) | 15 (45.5) | 10 (30.3) |
| Number of UVCs (median [IQR]) | 0.00 [0.00, 1.00] | 0.00 [0.00, 0.00] |
| UAC^4^ = yes (%) | 7 (21.2) | 8 (24.2) |
| Number of UACs (median [IQR]) | 0.00 [0.00, 0.00] | 0.00 [0.00, 0.00] |
| LL^5^ = yes (%) | 11 (33.3) | 10 (30.3) |
| *Vena iliaca (median [IQR])* | 1.00 [1.00, 1.00] | NA [NA, NA] |
| *Vena saphena magna (median [IQR])* | 1.00 [1.00, 1.00] | NA [NA, NA] |
| *Vena cephalica (median [IQR])* | 1.00 [1.00, 1.00] | 1.00 [1.00, 1.00] |
| *Vena basilica (median [IQR])* | 1.00 [1.00, 1.00] | 1.00 [1.00, 1.00] |
| *Vena brachialis (median [IQR])* | 1.00 [1.00, 1.00] | 1.00 [1.00, 1.00] |
| *Vena femoralis (median [IQR])* | 1.00 [1.00, 1.00] | 1.00 [1.00, 1.00] |
| *Vena fosa cubiti (median [IQR])* | 1.00 [1.00, 1.00] | NA [NA, NA] |
| *Unknown (median [IQR])* | 1.00 [1.00, 1.00] | 1.00 [1.00, 1.00] |
| Number of LLs (median [IQR]) | 0.00 [0.00, 1.00] | 0.00 [0.00, 1.00] |
| Arterial cannulation = yes (%) | 11 (33.3) | 4 (12.1) |
| *Arteria radialis (median [IQR])* | 2.00 [1.25, 2.00] | 1.00 [1.00, 1.00] |
| *Arteria brachialis (median [IQR])* | 1.00 [1.00, 1.00] | NA [NA, NA] |
| *Arteria femoralis (median [IQR])* | 2.50 [1.75, 3.25] | NA [NA, NA] |
| *Arteria ulnaris (median [IQR])* | NA [NA, NA] | 1.00 [1.00, 1.00] |
| *Unknown (median [IQR])* | 1.00 [1.00, 1.00] | 2.00 [2.00, 2.00] |
| Number of arteria cannulations (median [IQR]) | 0.00 [0.00, 1.00] | 0.00 [0.00, 0.00] |
| Capillary blood samples = yes (%) | 33 (100.0) | 33 (100.0) |
| Number of capillary blood samples (median [IQR]) | 8.00 [6.00, 23.00] | 10.00 [7.00, 16.00] |
| Total number of skin perforations (median [IQR]) | 16.00 [7.00, 35.00] | 13.00 [7.00, 22.00] |
| Surgery = yes (%) | 12 (36.4) | 4 (12.1) |
| Respiratory support |  |  |
| Intubation = yes (%) | 25 (75.8) | 11 (33.3) |
| Respirator = yes (%) | 19 (57.6) | 9 (27.3) |
| *Respirator days (median [IQR])* | 1.00 [0.00, 4.00] | 0.00 [0.00, 1.00] |
| CPAP^6^ = yes (%) | 28 (84.8) | 25 (75.8) |
| *CPAP days (median [IQR])* | 7.00 [2.00, 20.00] | 4.00 [1.00, 11.00] |
| HF^7^ = yes (%) | 14 (42.4) | 12 (36.4) |
| *HF days (median [IQR])* | 0.00 [0.00, 14.00] | 0.00 [0.00, 4.00] |
| NIV^8^ = yes (%) | 1 (3.0) | 1 (3.0) |
| *NIV days (median [IQR])* | 0.00 [0.00, 0.00] | 0.00 [0.00, 0.00] |
| Total number of days in respiratory support (median [IQR]) | 10.00 [3.00, 32.00] | 5.00 [1.00, 26.00] |
| Imaging |  |  |
| ECG = yes (%) | 6 (18.2) | 3 (9.1) |
| X-ray = yes (%) | 27 (81.8) | 16 (48.5) |
| CT = yes (%) | 2 (6.1) | 0 (0.0) |
| Ultrasound (US) = yes (%) | 15 (45.5) | 22 (66.7) |
| MR = yes (%) | 2 (6.1) | 0 (0.0) |
| Echocardiography = yes (%) | 15 (45.5) | 8 (24.2) |
| ECMO^9^ = yes (%) | 1 (3.0) | 0 (0.0) |
| Department characteristics |  |  |
| Number of fellow patients in room (median [IQR]) | 2.00 [1.00, 3.00] | 2.00 [1.00, 5.00] |
| Number of HCP contacts (median [IQR]) | 42.00 [24.00, 86.00] | 39.00 [28.00, 63.00] |
| Admitted to premature-unit first = yes (%) | 11 (33.3) | 2 (6.1) |
| Admitted to mature-unit = yes (%) | 24 (72.7) | 10 (30.3) |
| *Room 1 = yes (%)* | 6 (18.2) | 2 (6.1) |
| *Room 2 = yes (%)* | 6 (18.2) | 3 (9.1) |
| *Room 3 = yes (%)* | 5 (15.2) | 1 (3.0) |
| *Room 4 = yes (%)* | 9 (27.3) | 2 (6.1) |
| *Room 5 = yes (%)* | 4 (12.1) | 1 (3.0) |
| *Room 6 = yes (%)* | 10 (30.3) | 1 (3.0) |
| *Room 7 = yes (%)* | 3 (9.1) | 1 (3.0) |
| *Room 8 = yes (%)* | 3 (9.1) | 4 (12.1) |
| *Room 9 = yes (%)* | 7 (21.2) | 6 (18.2) |
| Admitted to premature-unit = yes (%) | 19 (57.6) | 24 (72.7) |
| *Room 10 = yes (%)* | 2 (6.1) | 3 (9.1) |
| *Room 11 = yes (%)* | 1 (3.0) | 4 (12.1) |
| *Room 12 = yes (%)* | 8 (24.2) | 5 (15.2) |
| *Room 13 = yes (%)* | 4 (12.1) | 2 (6.1) |
| *Room 14 = yes (%)* | 1 (3.0) | 2 (6.1) |
| *Room 15 = yes (%)* | 3 (9.1) | 5 (15.2) |
| *Room 16 = yes (%)* | 2 (6.1) | 4 (12.1) |
| *Room 17 = yes (%)* | 2 (6.1) | 9 (27.3) |
| Admitted to unit 7044 = yes (%) | 4 (12.1) | 2 (6.1) |
| *Room 444 = yes (%)* | 1 (3.0) | 2 (6.1) |
| *Room 448 = yes (%)* | 2 (6.1) | 1 (3.0) |
| *Room 452 = yes (%)* | 1 (3.0) | 1 (3.0) |
| Total number of rooms (median [IQR]) | 2.00 [1.00, 3.00] | 1.00 [1.00, 2.00] |
| Microbiological characteristics |  |  |
| Antibiotics |  |  |
| Antibiotics during admission = yes (%) | 23 (69.7) | 15 (45.5) |
| Number of antibiotics administrated (median [IQR]) | 9.00 [0.00, 30.00] | 0.00 [0.00, 15.00] |
| Number of different antibiotics (median [IQR]) | 2.00 [0.00, 4.00] | 0.00 [0.00, 3.00] |
| *Cefuroxime = yes (%)* | 7 (21.2) | 5 (15.2) |
| *Cefotaxime = yes (%)* | 3 (9.1) | 4 (12.1) |
| *Meropenem = yes (%)* | 6 (18.2) | 5 (15.2) |
| *Vancomycin = yes (%)* | 10 (30.3) | 3 (9.1) |
| *Gentamicin = yes (%)* | 17 (51.5) | 10 (30.3) |
| *Metronidazole = yes (%)* | 5 (15.2) | 4 (12.1) |
| *Benzylpenicillin = yes (%)* | 11 (33.3) | 3 (9.1) |
| *Piperacillin/Tazobactam = yes (%)* | 10 (30.3) | 9 (27.3) |
| *Amoxicillin = yes (%)* | 2 (6.1) | 2 (6.1) |
| *Cloxacillin = yes (%)* | 1 (3.0) | 2 (6.1) |
| MRSA-testing |  |  |
| Hospitalization days before positive test (median [IQR]) | 3.00 [0.00, 20.00] | 10.00 [5.00, 20.00] |
| Post menstrual age at first test (weeks) (median [IQR]) | 33.10 [31.10, 38.30] | 34.60 [31.20, 36.60] |
| Sampling site (%)^10^ |  |  |
| Screening samples |  |  |
| *Nose-throat-perineum^11^ = yes (%)* | 12 (36.4) | 1 (3.0) |
| *Nose = yes (%)* | 16 (48.5) | 0 (0.0) |
| *Throat = yes (%)* | 18 (54.5) | 0 (0.0) |
| *Perineum = yes (%)* | 15 (45.5) | 0 (0.0) |
| Clinical samples |  |  |
| *Wound = yes (%)* | 3 (9.1) | 3 (9.1) |
| *Sputum = yes (%)* | 5 (15.2) | 6 (18.2) |
| *Conjuctiva = yes (%)* | 6 (18.2) | 19 (57.6) |
| *Blood = yes (%)* | 4 (12.1) | 9 (27.3) |
| *Other = yes (%)* | 9 (27.3) | 4 (12.5) |

PVC^1^ = Peripheral venous catheter, CVC^2^ = Central venous catheter, UVC^3^ = Umbilical venous catheter, UAC^4^ = Umbilical artery catheter, LL^5^ = Long line, CPAP^6^ = Continuous positive airway pressure, HF^7^ = High flow, NIV^8^ = Non-invasive ventilation, ECMO^9^ = Extracorporeal membrane oxygenation, ^10^ Each infant can be included several times if the infant has been colonized in several places, ^11^ These are pooled samples, i.e., we do not know if the infant is positive 1, 2 or 3 places
